# Associations between ambient air pollutants and hospital admissions: more needs to be done

**DOI:** 10.1007/s11356-021-16544-0

**Published:** 2021-09-30

**Authors:** Jill JF Belch, Catherine Fitton, Bianca Cox, James D Chalmers

**Affiliations:** 1 Ninewells Hospital and Medical School, Dundee, DD1 9SY UK; 2 Leuven University: Katholieke Universiteit Leuven, Leuven, Belgium

**Keywords:** Air pollution, Cardiovascular disease, Hospital admissions

## Abstract

Deaths from air pollution in the UK are higher by a factor of 10 than from car crashes, 7 for drug-related deaths and 52 for murders, and yet awareness seems to be lacking in local government. We conducted an 18-year retrospective cohort study using routinely collected health care records from Ninewells Hospital, Dundee, and Perth Royal Infirmary, in Tayside, Scotland, UK, from 2000 to 2017. Hospitalisation events and deaths were linked to daily nitric oxides (NOX, NO, NO2), and particulate matter 10 (PM10) levels extracted from publicly available data over this same time period. Distributed lag models were used to estimate risk ratios for hospitalisation and mortality, adjusting for temperature, humidity, day of the week, month and public holiday. Nitric oxides and PM10 were associated with an increased risk of all hospital admissions and cardiovascular (CV) admissions on day of exposure to pollutant. This study shows a significant increase in all cause and CV hospital admissions, on high pollution days in Tayside, Scotland.

**Supplementary Information:**

The online version contains supplementary material available at 10.1007/s11356-021-16544-0.

## Introduction

Globally it is estimated that 16% of deaths are related to air pollution (Landrigan et al. 2018) with around 40,000 UK deaths/annum attributable to outdoor air pollution (Royal College of Physicians 2016) mostly from traffic. This extrapolates to 50 deaths a week occurring in Scotland (Royal College of Physicians 2016), the area of study. Air pollution plays a role in many health challenges and is projected by end 2031 to be the leading environmental cause of mortality globally (Royal College of Physicians 2016).

The general public find it difficult to believe that deaths from air pollution in the UK are 10 times that from car crashes (1,770 reported road deaths 2018 (National Records of Scotland 2020)), 7 times the numbers of drug-related deaths (5546 in 2018 (Office for National Statistics 2019; Statista 2020)) and 52 times the number of murders (701 England 2018/2019(Scottish Government 2018), 59 Scotland 2017 (Nawrot et al. 2011)). There is a frightening ignorance of the level of risk to health that occurs with air pollution, even in urban and small city regions. It is the most disadvantaged who suffer most, being frequently located near busy polluting roads.

Concentration limits for pollutants have been set by the UK and devolved governments, not by adhering to the EU Regulations (Eur-Lex 2008) but using the World Health Organization’s air quality guidelines (World Health Organization Europe 2006). The current WHO value of 40μg/m3 (annual mean) for nitric oxides (NO2) was set to protect the public from the health effects of gaseous NO2. The EU directive maximum level for annual values is 32 and 28μg/m3 for NOx and particulate matter (PM), respectively (Butland et al. 2017). Despite both the WHO (World Health Organization Europe 2006) and EU regulations (Eur-Lex 2008) (the latter is law), high levels of toxic pollution are still tolerated.

This study, from the Tayside Pollution Research Programme (TPRP), aims to investigate the effects of air pollution on acute hospital admissions over a 17-year period, across Tayside, Scotland, containing two of Scotland’s smaller cities: Dundee (population circa 148,270) and Perth (population circa 44,820).

## Materials and methods

### Patient population

A record linkage time series study of all hospital admissions at Ninewells Hospital, Dundee, and Perth Royal Infirmary, Perth, was carried out from January 1, 2000, to December 31, 2017. Consent was given by the Tayside Caldicott Guardian. As these data were anonymised, the requirement for consent to publish is not applicable.

### Data management

Unique personal identifier codes (CHI) were used to extract electronic medical records from a database for Tayside. All data storage and analyses were carried out on anonymised data, within the Safe Haven, the Tayside Health Informatics Centre (HIC), who provided the linkage of admissions to daily pollution levels across the region.

### Hospital admission and deaths definition

Hospital admissions of interest were agreed a priori and were defined by ICD10 code (2016 addition), identified from the Scottish Morbidity Record 01 (SMR01) database. These were all admissions regardless of ICD10 code, relevant cardiovascular admissions (ICD10 code: selected from within I00-99). Cardiovascular (CV) admissions were restricted to patients over 45 to exclude congenital disease. Deaths were identified from the National Records of Scotland (NRS).

### Exposure data

Daily PM10 (particulate matter with a diameter <10μm), NOX, nitrogen dioxide (NO2) and nitric oxide (NO) concentrations were measured during the study period at the Dundee and Perth urban background sites, (part of the UK’s Automatic Urban and Rural Network (AURN)). The risk of having a hospital admission or dying for each 10μg/m^3^ increase in PM10, NOX, NO2 and NOx was calculated. Highest quartiles and deciles were compared to the lowest. PM2.5μm data were excluded as these were only measured for 5 years in Dundee and not in Perth. Days with missing air pollution data were also excluded.

Because climate is a known confounder between air pollution and disease (Ye et al. 2012), data on mean air temperature and relative humidity were obtained. For temperature, data from the measuring stations Mylnefield (Lat, 56.45699; Long, −3.07182) and Dalwhinnie (Lat, 56.9333; Long, −4.2333) were used, for Dundee and Perth, respectively. Humidity data were recorded at Leuchars (Lat, 56.3833; Long, −2.8667) (8 miles distant) and were used for Dundee; for Perth, Dalwhinnie data were used.

### Statistical analysis

To account for delayed effects of air pollution on ALI admissions, we combined quasi-Poisson regression with distributed lag (non-linear) models (DL(N)Ms), using separate models for PM10, NOx, NO2 and NO. DL(N)Ms enable the investigation of the temporal pattern of the association, providing an estimate of the “overall” effect of the exposure, incorporating potential delayed and harvesting effects. A DL(N)M model is defined through a “cross-basis” function, a bi-dimensional space of functions describing simultaneously the shape of the relationship along the space of the predictor (exposure-response function) and its distributed lag effects (lag-response function). We used a natural spline non-linear exposure-response function for the association between air pollution exposure and hospital admissions/deaths**.** The number of days included in the cross-basis and degrees of freedom for the natural cubic splines were chosen using a combination of qAIC and visual inspection of the 3D exposure-lag-response surfaces. An extended lag period of 0–5 days (day of exposure up to 5 days after) was used for all, CVD only, heart failure (HF) and myocardial infarction (MI) admissions, and for mortality. The lag structure was modelled with a natural cubic spline with three degrees of freedom (df), placing the knots at equally spaced values on the log scale of lags to allow more flexible lag effects at shorter delays. Categorical variables for day of the week (1–7), month (1–12) and public holidays (0 or 1) were included in the model to control for any weekly or monthly patterns in ALI admission. To account for the (potentially delayed) effects of meteorological factors on ALI admissions (36), we also included DLNM cross-bases for mean temperature and for humidity in the model. In both cross-bases, the maximum lag was set to 14 days, and natural cubic splines with five df were used to model the exposure-response and the lag-response functions, respectively.

Risk ratios (RR) of hospital admissions and deaths were calculated for moderate (75th percentile) and high (95th percentile) exposure to air pollutant concentrations, compared to that of low (0 percentile) exposure (1s). Reported estimates, computed as the risk at day 0 (day of exposure), and the cumulative risk over the total lag period, are presented with corresponding 95% confidence intervals (CI).

All analyses were performed with the statistical software R (R Foundation for Statistical Computing, Vienna, Austria) using the “dlnm” package (https://cran.r-project.org/web/packages/dlnm/index.html).

## Results

Details about the admission breakdown and pollution levels over the study period are given in supplementary information (Tables 1s-5s, Fig 1s). Fig 2s-5s show the 3D plots and the exposure-response plots for the non-linear models. Figs 2s-5s show that the models are non-linear in nature, and the slope of the association is steeper with higher concentrations. The effects are seen on day of exposure rather than over the lag period, shown by an increased RR on day 0 and a return to RR 1.00 after lag 1 (Fig 2s-3s).

Moderate (P75 vs P0 exposure) and high (P95 vs P0 exposure) exposure to all pollutants was associated with an increased risk of hospital admission for any reason in Dundee and Perth on day of exposure (Table 1). This association was not reflected following the lag period (lag 0–5). Similarly, moderate and high exposure to all pollutants was associated with an increased risk of CVD hospital admissions on day of exposure in Dundee and Perth, but not following the lag period (Table 2).

**Table 1:** Adjusted risk ratios with 95% confidence intervals for all hospital admissions for adults >45years and exposure to pollutants in Dundee and Perth. Lag 0 and cumulative risk (lag 0–5 days) given. Results presented as moderate (P75 vs P0) or high (P95 vs P0) exposure to a pollutant

Exposure	Dundee		Perth	
	Moderate exposure (P75 vs P0)	High exposure (P95 vs P0)	Moderate exposure (P75 vs P0)	High exposure (P95 vs P0)
Lag 0				
PM10	1.103 (1.052–1.157)	1.127 (1.074–1.182)	1.388 (1.267–1.521)	1.438 (1.315–1.572)
NOX	2.083 (1.958–2.216)	2.281 (2.142–2.429)	1.543 (1.455–1.637)	1.653 (1.556–1.756)
NO2	1.954 (1.806–2.114)	2.093 (1.933–2.266)	1.490 (1.389–1.598)	1.581 (1.471–1.701)
NO	2.255 (2.026–2.509)	2.343 (2.017–2.723)	1.520 (1.442–1.601)	1.625 (1.539–1.715)
Cumulative				
PM10	1.028 (0.985–1.073)	1.020 (0.978–1.064)	0.976 (0.901–1.057)	0.970 (0.898–1.049)
NOX	0.953 (0.910–0.998)	0.956 (0.913–1.001)	0.946 (0.902–0.989)	0.941 (0.897–0.989)
NO2	0.976 (0.918–1.037)	0.973 (0.915–1.035)	0.957 (0.905–1.013)	0.959 (0.904–1.018)
NO	0.944 (0.859–1.039)	0.913 (0.790–1.055)	0.942 (0.904–0.983)	0.937 (0.897–0.980)

**Table 2:** Adjusted risk ratios with 95% confidence intervals for all CV hospital admissions of patients under > 45 years, in Dundee and Perth and exposure to pollutants. Lag 0 and cumulative risk (lag 0–5 days) given. Results presented as moderate (P75 vs P0) or high (P95 vs P0) exposure to a pollutant

Exposure	Dundee		Perth	
	Moderate exposure (P75 vs P0)	High exposure (P95 vs P0)	Moderate exposure (P75 vs P0)	High exposure (P95 vs P0)
Lag 0				
PM10	1.061 (0.998−1.128)	1.076 (1.013−1.033)	1.394 (1.225−1.585)	1.449 (1.278−1.643)
NOX	1.941 (1.785−2.111)	2.057 (1.889−2.240)	1.589 (1.460−1.729)	1.707 (1.565−1.862)
NO2	1.816 (1.638−2.014)	1.894 (1.706−2.103)	1.528 (1.383−1.688)	1.650 (1.488−1.829)
NO	1.901 (1.763−2.051)	1.942 (1.802−2.094)	1.552 (1.440−1.673)	1.654 (1.530−1.789)
Cumulative				
PM10	0.998 (0.946−1.054)	0.979 (0.927−1.033)	0.999 (0.893−1.118)	0.988 (0.886−1.101)
NOX	0.947 (0.889−1.008)	0.951 (0.893−1.012)	0.944 (0.883−0.989)	0.922 (0.859−0.989)
NO2	0.946 (0.874−1.024)	0.950 (0.876−1.029)	0.940 (0.867−1.018)	0.944 (0.867−1.027)
NO	0.953 (0.901−1.008)	0.953 (0.902−1.007)	0.945 (0.890−1.004)	0.918 (0.861−0.978)

There was an association between moderate and high pollutant exposure and MI hospital admission across Dundee and Perth on day of exposure; however, results were only significant in the Perth location (Table 3). There was no clear association following the lag period. Moderate and high pollutant exposure was associated with increased HF hospital admissions on day of exposure in Dundee and Perth; however, this again was only significant for Perth (Table 4). There was also no clear association with risk of admission following the lag period.

**Table 3:** Adjusted risk ratios with 95% confidence intervals for MI hospital admissions of patients under > 45 years, in Dundee and Perth and exposure to pollutants. Lag 0 and cumulative risk (lag 0–5 days) given. Results presented as moderate (P75 vs P0) or high (P95 vs P0) exposure to a pollutant

Exposure	Dundee		Perth	
	Moderate exposure (P75 vs P0)	High exposure (P95 vs P0)	Moderate exposure (P75 vs P0)	High exposure (P95 vs P0)
Lag0				
PM10	1.048 (0.917–1.198)	1.055 (0.922–1.067)	1.158 (0.885–1.514)	1.164 (0.895–1.512)
NOX	1.178 (0.988–1.405)	1.199 (1.000–1.438)	1.484 (1.237–1.780)	1.527 (1.265–1.844)
NO2	1.136 (0.918–1.406)	1.140 (0.916–1.418)	1.506 (1.213–1.869)	1.550 (1.239–1.941)
NO	1.147 (0.977–1.346)	1.158 (0.987–1.357)	1.456 (1.238–1.712)	1.498 (1.264–1.776)
Cumulative				
PM10	0.973 (0.863–1.098)	0.947 (0.840–1.067)	1.111 (0.870–1.419)	1.104 (0.870–1.399)
NOX	0.907 (0.786–1.048)	0.886 (0.767–1.023)	1.031 (0.887–1.204)	1.031 (0.882–1.204)
NO2	0.926 (0.773–1.107)	0.928 (0.775–1.111)	1.030 (0.861–1.232)	1.023 (0.848–1.234)
NO	0.902 (0.792–1.027)	0.890 (0.783–1.011)	1.013 (0.885–1.160)	1.012 (0.879–1.165)

**Table 4:** Adjusted risk ratios with 95% confidence intervals for HF hospital admissions of patients under > 45 years, in Dundee and Perth and exposure to pollutants. Lag 0 and cumulative risk (lag 0–5 days) given. Results presented as moderate (P75 vs P0) or high (P95 vs P0) exposure to a pollutant

Exposure	Dundee		Perth	
	Moderate exposure (P75 vs P0)	High exposure (P95 vs P0)	Moderate exposure (P75 vs P0)	High exposure (P95 vs P0)
Lag0				
PM10	1.032 (0.884–1.205)	1.112 (0.952–1.265)	1.569 (1.098–2.243)	1.667 (1.176–2.362)
NOX	1.960 (1.580–2.430)	2.117 (1.701–2.634)	1.772 (1.389–2.262)	1.867 (1.456–2.394)
NO2	1.933 (1.480–2.524)	2.057 (1.571–2.692)	1.583 (1.191–2.104)	1.693 (1.261–2.274)
NO	1.885 (1.552–2.288)	1.929 (1.591–2.338)	1.689 (1.361–2.096)	1.752 (1.402–2.190)
Cumulative				
PM10	1.086 (0.944–1.249)	1.101 (0.958–1.265)	0.956 (0.700–1.306)	0.981 (0.725–1.327)
NOX	0.910 (0.777–1.065)	0.909 (0.775–1.065)	0.970 (0.801–1.143)	0.933 (0.762–1.143)
NO2	0.968 (0.793–1.183)	0.969 (0.792–1.186)	1.010 (0.800–1.275)	1.021 (0.799–1.303)
NO	0.891 (0.774–1.026)	0.889 (0.774–1.021)	0.969 (0.816–1.151)	0.935 (0.778–1.123)

All-cause mortality was not associated with any pollution exposure, in Dundee or Perth, for either day of exposure or cumulative exposure (Table 6s).

## Discussion

This 17-year time series analysis study shows that air pollution is associated with an increase of all hospital admissions, and CVD admissions, for adults >45years in two small cities in Scotland on day of exposure to pollution. We also report an association between pollution exposure with MI and HF hospital admissions, on day of exposure; however, significance was not reached for all these results. We did not find a risk of increased hospital admissions for any admission type over the cumulative lag period. Furthermore, we did not find an association between pollution exposure and recorded deaths during the study period.

This work is novel in that other publications, showing similar adverse effects of pollution, report major cities with population counts in the millions (Dockery et al. 1993; Goeminne et al. 2018; Butland et al. 2017). This work adds to the literature by showing that smaller cities (population 50k–150k) can still suffer significant, and measurable, adverse effects of air pollution. This is not well recognised with 98% of low emissions zones in the UK being in its large major cities (Department for Environment Food and Rural Affaires and Department of Transport 2020).

The health problems resulting from exposure to air pollution also have a high financial cost to society and, as shown by our results, our health services. In the UK, these NHS costs add up to more than £20 billion every year (Royal College of Physicians 2016). Vulnerable/disadvantaged people are prisoners of air pollution, as they live in its midst. It is surprising that local authorities do not seem to have connected social and health care costs with pollution and realised that the added cost spent on preventing/ameliorating pollution will pay for itself in terms of social and health care down the line (Holland 2014).

Air pollution, particularly from external sources, is today’s “silent killer”. And its silent nature means little action is being taken. Providing more evidence for links to ill-health will hopefully provide the Scottish Government with the incentive enforcing their regulation by law. However, until it is enforced downwards, Councils with many calls on finances will remain unwilling to act, and hospital admissions will continue to reflect this cavalier attitude. The Cleaner Air for Scotland Strategy has much to recommend it but lacks enforcement teeth (Scottish Government 2015).

In conclusion, this paper further shows a harmful effect of pollution on hospital admissions, in two relatively small cities. It adds further evidence of the harm of air pollution, even in smaller UK cities. It reminds us also that as clinicians we should be using all our skills to attenuate this problem, as the Royal College of Physicians and the Royal College of Paediatricians, UK (Royal College of Physicians 2016), tell us:

“When our patients are exposed to such a clear and avoidable cause of death, illness and disability, it is our duty as doctors to speak out”.

## Supplementary information

ESM 1 (DOCX 406 kb)
