# Supplementary material for: Associations between ambient air pollutants and hospital admissions: more needs to be done
Source: Environ Sci Pollut Res Int. 2021 Sep 30;28(43):61848–52. doi: 10.1007/s11356-021-16544-0 (PMC8580915; doi:10.1007/s11356-021-16544-0)
Supplement: Supplementary file 1 — (DOCX 406 kb) [file 11356_2021_16544_MOESM1_ESM.docx]

**SUPLEMENTARY INFORMATION**

**Evidence Pollution Increases Hospital Admissions:**

**More Needs to be Done.**

**Jill JF Belch^1^, Catherine Fitton^1^, Bianca Cox^2^, James Chalmers^1^,**

**^1^University of Dundee NHS Tayside, Ninewells Hospital, Dundee DD1 9SY, ^2^Centre for Environmental Sciences, Hasselt University, Diepenbeek, Belgium.**

Table 1s. Values for P0, P75 and P95 for each pollutant/city

| Pollutant | P0 value | P75 value | P95 value |
| --- | --- | --- | --- |
| Dundee | | | |
| PM10 | 1.0 | 19.6 | 34.4 |
| NOX | 11.3 | 212 | 313.25 |
| NO2 | 7.8 | 60.2 | 76.125 |
| NO | 1.7 | 99.75 | 155.0 |
| Perth | | | |
| PM10 | 1.0 | 27.7 | 40.2 |
| NOX | 18.1 | 182.0 | 282.4 |
| NO2 | 11.0 | 64.5 | 85.74 |
| NO | 5.4 | 76.9 | 131.3 |

**Table 2. Proportion of hospital admission types in Dundee, split for adult and child admissions**

| **Admission diagnosis** | **Dundee - Proportion (%)** | | **Perth – Proportion (%)** | |
| --- | --- | --- | --- | --- |
|  | Adult | Under 16s | Adult | Under 16s |
| Circulation/Heart | 16.99 | 0.75 | 17.56 | 0.87 |
| Infectious/Parasitic Disease | 1.66 | 9.37 | 1.39 | 7.82 |
| Neoplasms | 15.93 | 5.00 | 19.67 | 4.57 |
| Endocrine/Metabolic | 2.08 | 1.31 | 1.83 | 1.21 |
| Nervous System | 2.56 | 2.31 | 2.43 | 2.40 |
| Eye/Ear | 4.37 | 3.55 | 4.21 | 3.62 |
| Respiratory | 9.98 | 17.01 | 7.47 | 16.71 |
| Digestive System | 9.98 | 16.49 | 10.13 | 18.22 |
| Skin/Subcutaneous Tissue | 1.89 | 2.14 | 1.65 | 1.95 |
| Musculoskeletal | 5.15 | 2.41 | 5.30 | 2.36 |
| Genitourinary | 6.44 | 3.52 | 5.94 | 4.75 |
| Congenital Malformations | 0.18 | 4.64 | 0.16 | 5.08 |
| Injury/Poisoning | 4.67 | 6.53 | 5.24 | 7.59 |
| Abnormal Clinical Findings | 10.95 | 10.79 | 10.17 | 12.46 |
| External Causes Of Morbidity | 2.79 | 3.21 | 2.80 | 3.11 |
| Others | 4.38 | 10.97 | 4.05 | 7.28 |

**Table 3s. Breakdown of ICD10 codes for circulatory admissions (ICD10 “I”), and the proportion of the group. Those used for the study marked with ***

| **ICD10 code** | **Description** | **Proportion (%)** |
| --- | --- | --- |
| I00-I02 | Acute rheumatic fever | 0.01 |
| I05-I09 | Chronic rheumatic heart diseases | 0.6 |
| I10-I15 | Hypertensive diseases* | 0.8 |
| I20-I25 | Ischaemic heart diseases* | 37.8 |
| I26-I28 | Pulmonary heart disease and diseases of pulmonary circulation | 3.2 |
| I30-I52 | Heart disease* | 26.4 |
| I60-I69 | Cerebrovascular diseases* | 16.3 |
| I70-I79 | Diseases of arteries, arterioles and capillaries* | 8.4 |
| I80-I89 | Diseases of veins, lymphatic vessels and lymph nodes, not elsewhere classified | 4.5 |
| I95-I99 | Other and unspecified disorders of the circulatory system | 1.7 |

*Data used in the CVD analyses

**Table 4s. Average admissions per day of year for Dundee and Perth.**

| Year | Average admins per day of year | |
| --- | --- | --- |
|  | Dundee | Perth |
| 2000 | 137.8 | 63.8 |
| 2001 | 129.3 | 59.6 |
| 2002 | 122.3 | 58.5 |
| 2003 | 124.8 | 62.5 |
| 2004 | 127.6 | 61.9 |
| 2005 | 127.5 | 61.7 |
| 2006 | 125.3 | 65.3 |
| 2007 | 125.9 | 62.8 |
| 2008 | 133.5 | 65.0 |
| 2009 | 130.9 | 60.3 |
| 2010 | 125.2 | 58.6 |
| 2011 | 125.8 | 58.8 |
| 2012 | 131.9 | 60.5 |
| 2013 | 135.5 | 58.5 |
| 2014 | 136.6 | 58.5 |
| 2015 | 135.3 | 60.7 |
| 2016 | 142.1 | 63.8 |
| 2017 | 141.8 | 62.7 |

**Table 5s. Mean, median and range for pollution in Dundee and Perth. Pollution is measured as** **ug/m3**

| **Range** | **PM10** | **NOX** | **NO2** | **NO** |
| --- | --- | --- | --- | --- |
| **Dundee** | | | | |
| Mean | 15.8 | 172.3 | 50.4 | 79.8 |
| Median | 13.4 | 165.0 | 49.7 | 75.0 |
| Range | 1.0-93.6 | 11.3-713.0 | 7.8-150.0 | 1.7-367.0 |
| **Perth** | | | | |
| Mean | 22.8 | 145.9 | 53.1 | 60.7 |
| Median | 21.0 | 134.0 | 52.0 | 52.9 |
| Range | 1.0-105.0 | 18.1-615.0 | 11.0-136.0 | 5.4-323.0 |

**Table 6s. Total hospital admission types split for time of year**

|  | **Total** | **CVD** |
| --- | --- | --- |
| **Admissions in Dundee** |  |  |
| April - September | 443,110 | 61,842 |
| October -March | 447,368 | 61,574 |
| **Admissions in Perth** |  |  |
| April - September | 205,379 | 30,444 |
| October -March | 210,053 | 30,929 |


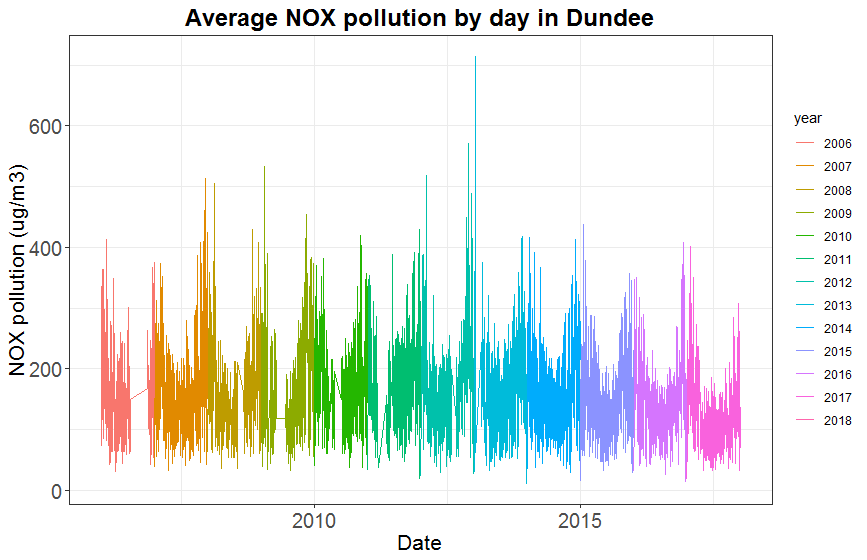


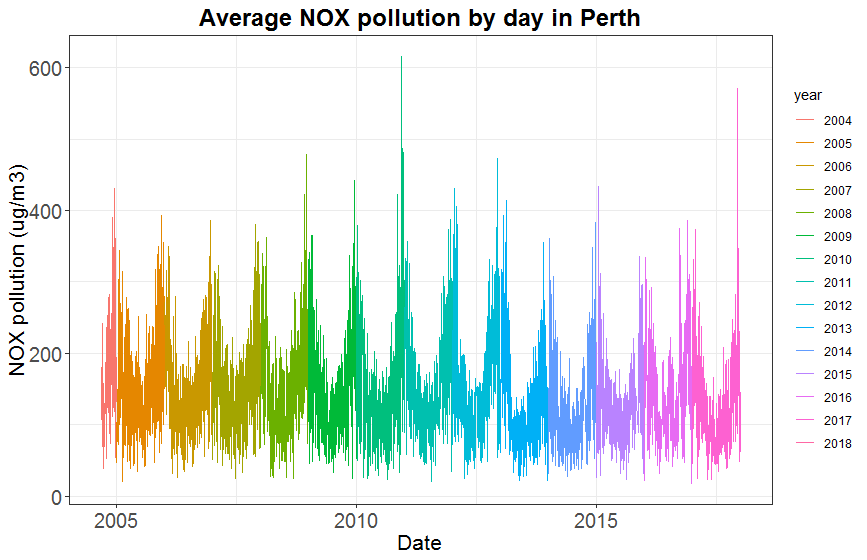


**Figure 1s: Average NOX levels per day in Perth and Dundee**


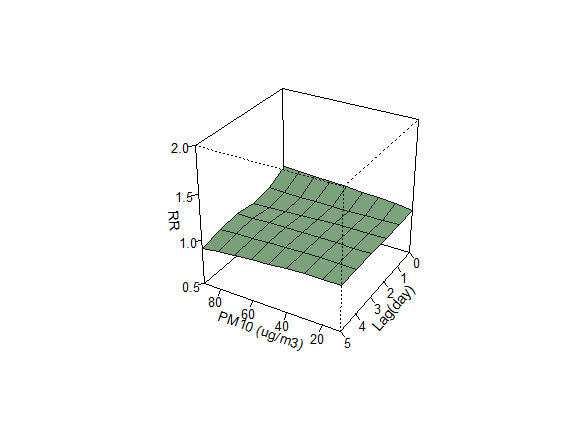

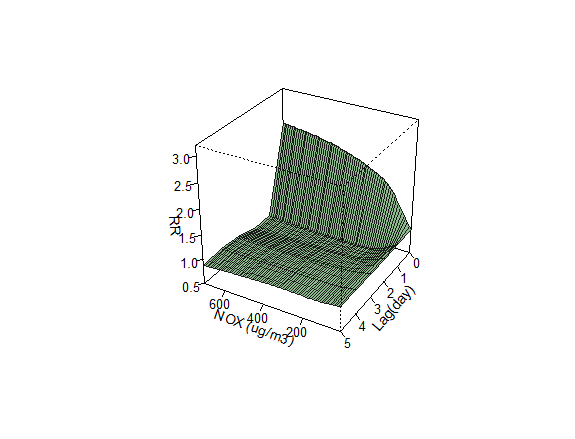


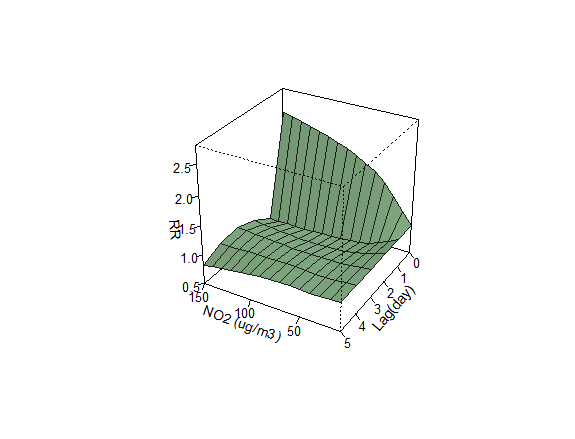

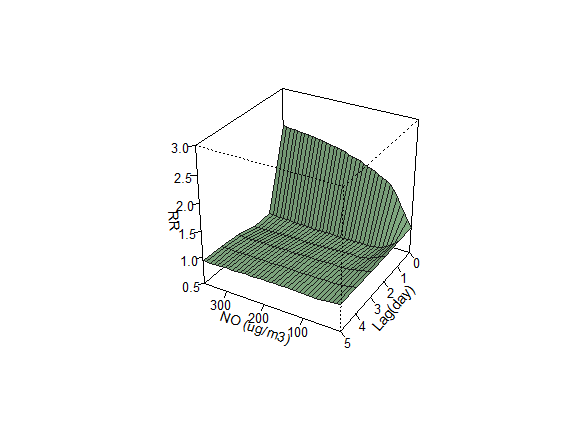


Fig 2s. 3D plots of the exposure-response surface, for all hospital admissions and PM10, NOX, NO2 and NO pollutants in Dundee


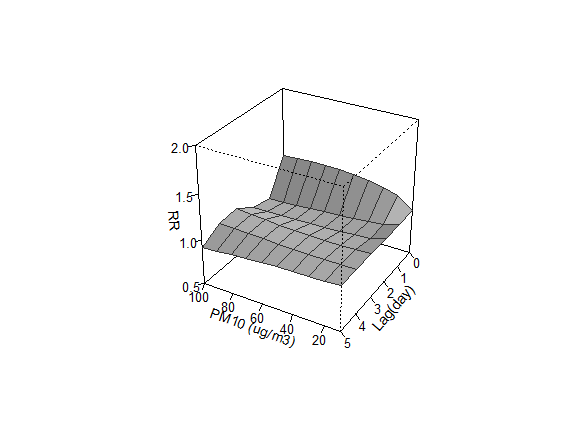

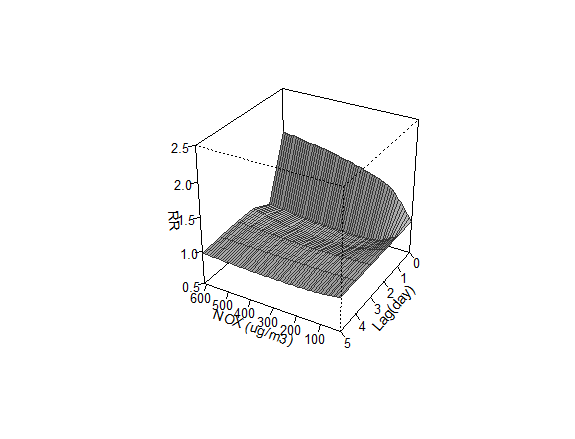


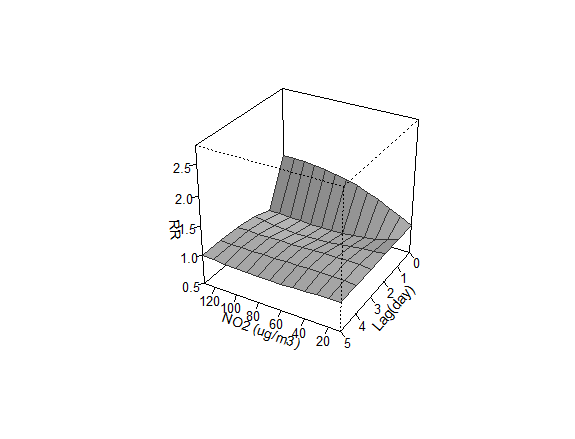

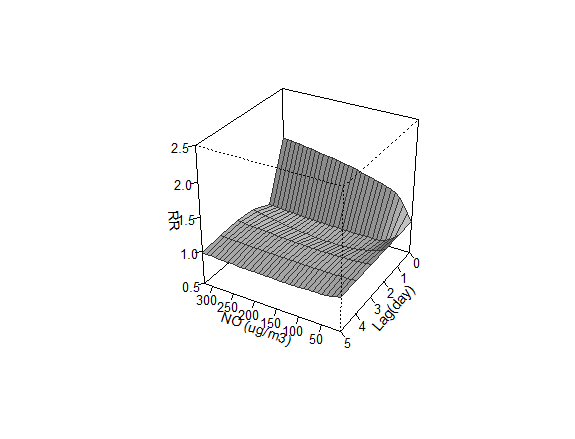


Fig 3s. 3D plots of the exposure-response surface, for all hospital admissions and PM10, NOX, NO2 and NO pollutants in Perth


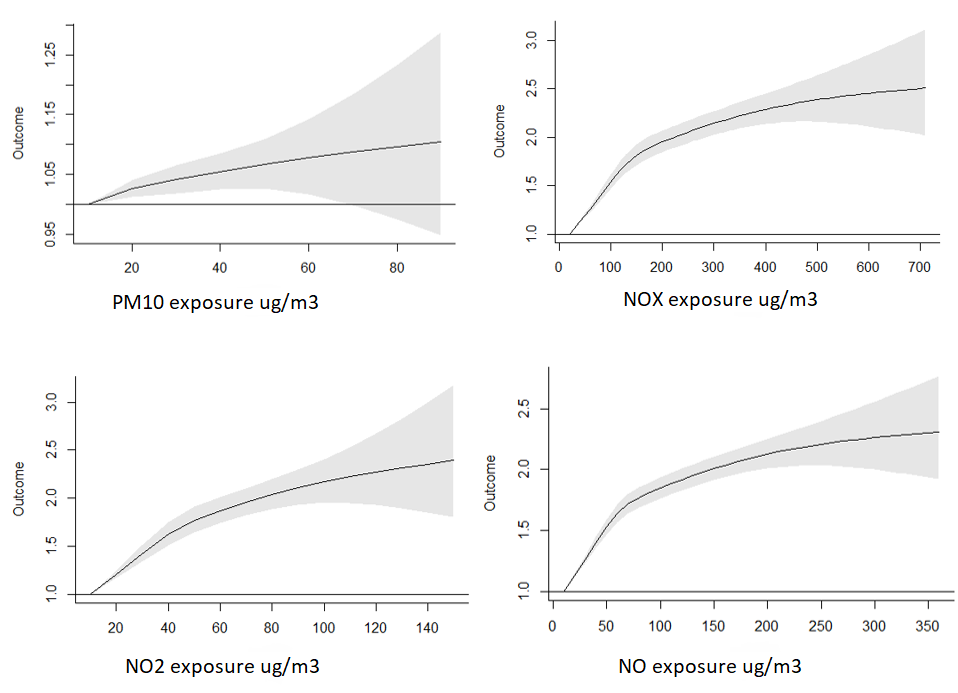


Fig 4s. Exposure-response plots for lag 0; for all hospital admissions and PM10, NOX, NO2 and NO pollutants in Dundee. Outcome is displayed as RR with 95% Cis.


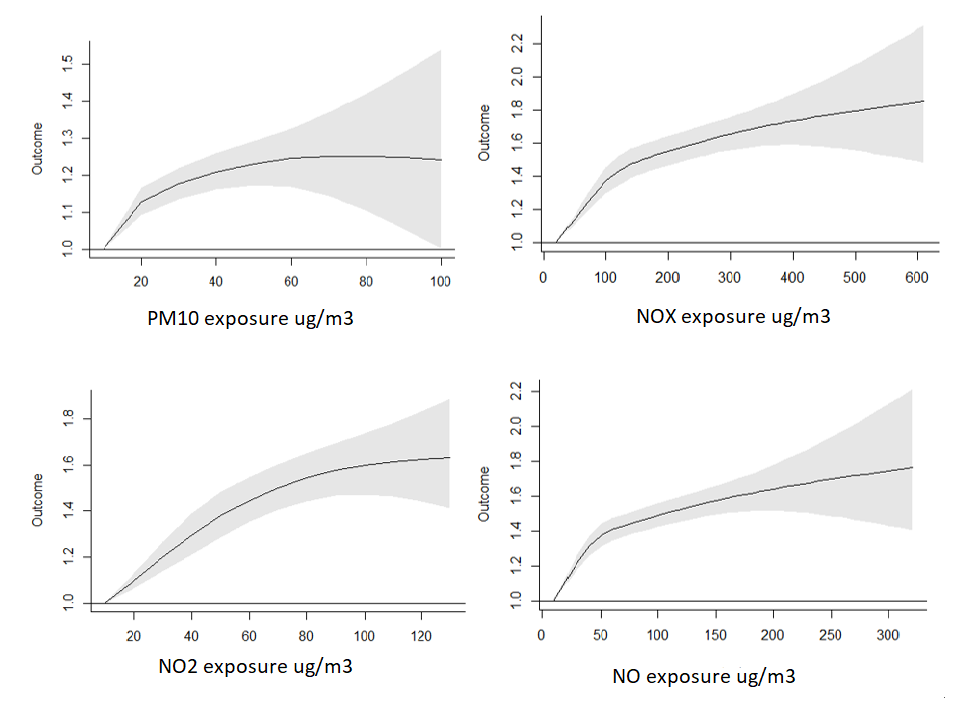


Fig 5s. Exposure-response plots for lag 0; for all hospital admissions and PM10, NOX, NO2 and NO pollutants in Perth. Outcome is displayed as RR with 95% Cis.

**Table 7s. Adjusted risk ratios with 95% confidence intervals for all-cause mortality in Dundee and Perth and exposure to pollutants. Results presented as moderate (P75 vs P0) or high (P95 vs P0) exposure to a pollutant**

| Exposure | Dundee | | Perth | |
| --- | --- | --- | --- | --- |
|  | Moderate exposure  (P75 vs P0) | High exposure  (P95 vs P0) | Moderate exposure  (P75 vs P0) | High exposure  (P95 vs P0) |
| Lag0 | | | | |
| PM10 | 0.970 (0.906-1.039) | 0.952 (0.889-1.045) | 0.857 (0.739-0.994) | 0.884 (0.765-1.021) |
| NOX | 1.020 (0.937-1.110) | 0.993 (0.910-1.083) | 1.004 (0.912-1.105) | 0.990 (0.896-1.095) |
| NO2 | 1.054 (0.952-1.167) | 1.025 (0.923-1.139) | 0.999 (0.892-1.119) | 0.997 (0.886-1.123) |
| NO | 1.002 (0.928-1.082) | 0.998 (0.924-1.077) | 0.996 (0.914-1.085) | 0.984 (0.898-1.078) |
| Cumulative | | | | |
| PM10 | 0.990 (0.931-1.053) | 0.984 (0.925-1.045) | 1.096 (0.952-1.261) | 1.077 (0.940-1.234) |
| NOX | 0.942 (0.877-1.011) | 0.939 (0.875-1.009) | 1.064 (0.976-1.133) | 1.037 (0.948-1.133) |
| NO2 | 0.949 (0.869-1.038) | 0.944 (0.863-1.032) | 1.059 (0.956-1.173) | 1.031 (0.926-1.148) |
| NO | 0.938 (0.880-1.000) | 0.941 (0.883-1.002) | 1.063 (0.984-1.148) | 1.039 (0.959-1.126) |
